# Comparison of next generation sequencing, SNaPshot assay and real-time polymerase chain reaction for lung adenocarcinoma EGFR mutation assessment

**DOI:** 10.1186/s12890-016-0250-0

**Published:** 2016-05-23

**Authors:** Andrei-Tudor Cernomaz, Ina Iuliana Macovei, Ionut Pavel, Carmen Grigoriu, Mihai Marinca, Florent Baty, Simona Peter, Radu Zonda, Martin Brutsche, Bogdan- Dragos Grigoriu

**Affiliations:** Department of Thoracic Oncology, Regional Institute of Oncology, University of Medicine and Pharmacy Iasi, Str. Gen. Berthelot, 2-4, Iasi, 700384 Romania; Lung Cancer Molecular Diagnostic Laboratory, University of Medicine and Pharmacy Iasi, Iasi, Romania; Pathology Department, University of Medicine and Pharmacy Iasi, Iasi, Romania; Oncology Department, Regional Institute of Oncology, University of Medicine and Pharmacy Iasi, Iasi, Romania; Department of Pulmonary Medicine, Cantonal Hospital St. Gallen, St. Gallen, Switzerland

## Abstract

**Background:**

The epidermal growth factor receptor (EGFR) mutation status assessment has become increasingly important given the significant impact of tyrosine kinase inhibitors in lung cancer management.

Our aim was to compare real life operational characteristics for three EGFR mutation assays - two targeted approaches and a next generation sequencing (NGS) technique.

**Methods:**

EGFR mutation status was assessed for lung adenocarcinoma samples (formalin fixed- paraffin embedded samples) using qPCR, SNaPshot and NGS (Ion Torrent™) techniques.

**Results:**

A total of 15 high clinical significance mutations were identified by at least one technique from the total of 64 samples. All mutations were identified by the TaqMan qPCR technique while SNaPshot in conjunction with fragment analysis identified 11 EGFR mutations. The NGS approach followed by an automatic analysis using the default calling parameters identified 10 mutations from the SNaPshot/qPCR panel and other three insertions, five point mutations and 58 silent variants; manual data review identified all 15 high significance mutations.

**Conclusions:**

Performance was similar for high tumor content samples but careful data analysis and post hoc variant calling filter alterations were necessary in order to obtain robust results from low tumor content samples by NGS. NGS is able to generate a comprehensive mutational profile albeit at a higher cost and workload. Result interpretation should take into account not only general run parameters such as mean read depth but also relative coverage and read distribution; currently there is an acute need to define firm recommendations/standards concerning NGS data interpretation and quality control.

## Background

Epidermal growth factor receptor (EGFR) mutation status is regarded as a particularly important element for improving non squamous non-small cell lung cancer (NSCLC) prognosis. EGFR mutations were reported with a frequency of around 10 % for total lung adenocarcinoma but up to 40 % in some Asian cohorts – mainly represented by exon 19 deletions and one exon 21 point substitution *c.2369C > T (L858R)* [1, 2]. Both lead to ligand-independent activation of the tyrosine kinase domain and confer sensitivity to EGFR tyrosine kinase inhibitors (TKIs). Other mutations were reported in less than 5 % of total cases - mainly point mutations *G719X* [3], *T783A, V765A* [4], and exon 20 insertions; despite low incidence they are assessed on a regular basis as some have been linked to response to EGFR TKIs. Mutation status can be determined by either targeted approaches or direct sequencing. Targeted approaches are used to detect a limited number of clinically significant mutations – usually therapy response predictors. These methods have higher sensitivity than Sanger sequencing and may reliably be used for small biopsy or cytology samples (with down to 1 % tumor cells content) [5].

Sequencing techniques have the main advantage of being able to identify all mutations in the studied region (previously known or not); main drawbacks are generally a higher workload and variable sensitivity – samples with tumor cells content over 20 % being usually required at least for Sanger technique. [6].

Currently there is no consensus on the optimal approach and existing guidelines do not strongly favor one method in particular.

Our aim was to compare real life operational characteristics for three EGFR mutation assays - two targeted approaches and a next generation sequencing (NGS) technique.

## Methods

Lung adenocarcinoma samples addressed for routine EGFR testing to the local molecular diagnostic laboratory between October 2013 and June 2015 were considered. Selection was based on availability of a previously signed informed consent allowing for biopsy samples banking and future research usage and availability of sufficient biological material without compromising further analyses if necessary.

Samples were anonymized prior to processing. Study protocol was reviewed and approved by the University of Medicine and Pharmacy “Grigore T. Popa” Iasi Ethics Commission (the 4th of August 2015).

Tissue samples were mainly obtained from primary tumors; there was one pleural fluid sample. Every sample was reviewed and tumor cell percentage was estimated by pathologists prior to DNA extraction; macro-dissection was performed if deemed necessary to increase tumor cell content.

Each sample underwent genomic DNA extraction using the Macherey Nagel FFPE DNA kit according to manufacturer specifications. DNA quality and quantity were assessed on an Eppendorf BioPhotometer Plus using a Helma Tray cell with 1 mm light path lid. EGFR mutation status was assessed using three independent methods – quantitative PCR (qPCR), SNaPshot assay, NGS.

For the primer extension reaction (SNaPshot) a multiplex PCR (GoTaq G2 Hot Start, Promega, Madison, WI, USA) was performed on 50 ng extracted DNA using primers and conditions from Table 1 [7]. PCR products were visualized by agarose gel electrophoresis to confirm correct amplification followed by enzymatic purification. Then one step extension was conducted following manufacturers recommendations and products were run on ABI PRISM 310 Genetic Analyzer (Life Technologies/Applied Biosystems, Foster City, CA, USA). EGFR exon 19 deletions were assessed using the method described by Pan et al. [8]. Data analysis was performed with GeneMapper Analysis Software version 4.0 (Life Technologies/Applied Biosystems) using the automatic calling parameters.

**Table 1:** Primer sequences and cycling details for multiplex PCR and SNaPshot

Primer	Primer sequence	Amplification conditions
	Multiplex PCR - Primers and Cycling Conditions	
EGFR Ex 18 FWD	5′- CCCCCCCAGCTTGTGG -3′	95^O^C - 3 min 45 cycles of: 94^O^C - 30 s 63^O^C - 90 s 72^O^C - 90 s Final extension 72^O^C - 10 min 4 ^O^C - end
EGFR Ex 18 REV	5′- ACCGTGCCGAACGCAC -3′	
EGFR Ex 20 FWD	5′- CTCTCCCTCCCTCCAGGAAG -3′	
EGFR Ex 20 REV	5′- TTCCCGGACATAGTCCAGGA -3′	
EGFR Ex 21 FWD	5′- CGCAGCATGTCAAGATCACAG -3′	
EGFR Ex 21 REV	5′- TGGCTGACCTAAAGCCACCT -3′	
	SNaPShot Assay - Primers and Cycling Conditions	
EGFR-2155R	5′- TGGTTAGATGGAACGCACCGGAGC -3′	96^O^C - 10 s 25 cycles of: 50^O^C 5 s 60^O^C - 30 s Final: 4^O^C - ∞ min
EGFR-2156R	5′- TAGATGTGGTTAGATGCGAACGCACCGGAG -3′	
EGFR-2369R	5′- TGGTTAGATGTGGTTAGATGGAAGGGCATGAGCTGC -3′	
EGFR-2573 F	5′- ATGTGGTTAGATGTGGTTAGATGAAGATCACAGATTTTGGGC -3′	
EGFR-2582 F	5′- GATGTGGTTAGATGTGGTTAGATGTGGTTAGATGTGGGCTGGCCAAAC -3′	
	Sizing Assay - Primers and Cycling conditions	
EGFR Ex19 FWD	5′ - GCACCATCTCACAATTGCCAGTTA-3	98^O^C - 3 min 45 cycles of: 98^O^C - 30 s 60^O^C - 60 s 72^O^C - 60 s Final extention: 72^O^C 10 min 4^O^C - ∞ min
EGFR Ex19 REV	5′-/6FAM/AAAAGGTGGGCCTGAGGTTCA-3)	

Quantitative PCR was performed using the EGFR Entrogen® kit on an AB 7500 Real-Time PCR system following manufacturer instructions. This kit usually requires 80 ng extracted DNA being able to detect 29 mutations: 19 different exon 19 deletions, exon 18 mutations *(G719X)* and 3 exon 20 insertions, *c.2361G > A (T790M), c.2369C > T (L858R), c.2582 T > A (L861Q), c.2303G > T (S768I)*.

Targeted NGS was performed on an Ion Torrent platform using the Ion AmpliSeq Cancer Hotspot Panel v2 (both from Life Technologies). Briefly, DNA amplification started from 10 ng of extracted DNA followed by barcode library construction using the Ion Ampliseq Library Kit 2.0 (Life Technologies). Following quantification, libraries were equalized at 100 pM, attached and amplified on Ion Sphere Particles. Sequencing was performed using PGM seq 200 kit v2 or PGM Hi-Q kit by pooling 6-8 libraries on an Ion 316 chip v2 and 10 - 11 libraries on an Ion 318 chip v2 according to manufacturer’s protocol. Aimed read depth was 1000×.

Sequence readings were aligned against the hg19 human genome, reference sequence NM_005228.3, using the default alignment settings (Ion Torrent Suite Software v 4.6). Data was analyzed using SEQUENCE Pilot module SeqNext v 4.2.0 (JSI Medical System); only EGFR data were considered; variant frequency threshold was set to 5 % with a minimum of 5 readings per strand for the automatic analysis as is recommended for AmpliSeq [9]; automatic calling was followed by manual review.

Price estimates were based on consumables cost alone; manpower costs were set aside as these figures may significantly differ between countries and settings (ex. clinical versus clinical research versus academic research). For NGS additional sample reruns (mainly for low coverage issues) were considered in the final estimate.

Numeric data is presented as mean +/- standard deviation.

## Results

Study pool contained 64 samples from 41 male and 23 female lung cancer patients; mean age was 62 +/- 9 years.

All samples were formalin-fixed paraffin-embedded (FFPE) - 36 were obtained from surgical specimens, 26 from small specimens (25 bronchial biopsies and one transthoracic core biopsy sample). There were two cytological samples - pleural fluid and transbronchial fine needle aspirate (upper lobe mass) cytoblocks. Macrodissection was deemed necessary and performed for 17 samples, nine being surgical specimens. Final estimated tumor cell percentage ranged between 1 and 75 % (mean 31 +/- 25 %).

Extracted DNA quantity was similar for both surgical and bronchial biopsies (8.7 +/- 9.1 ng/μl vs 7.1 +/- 7.1 ng/μl) despite larger volume and higher percentage of tumor cells per section for surgical samples (43 +/- 24 % vs 18 +/- 17 %). DNA quality estimated by the 260/280 ratio was 1.66 +/-0.28.

Quality control for NGS technique showed a mean mapped sequences of 312986 reads per sample and a mean read length of 105 +/- 7 bp. Average mean read depth was 1265 +/- 637× with a median of 1204× and interquartile range of 845 – 1680 × .

A total of 15 high clinical significance mutations were identified by at least one technique from a total of 14 samples. All mutations were identified by the TaqMan qPCR technique while SNaPshot in conjunction with fragment analysis identified 11 EGFR mutations (10 samples); one sample harbored two mutations - *c.2369C > T (L858R)* and *c.2361G > A (T790M)*.

A comprehensive view of EGFR mutation spectrum is showed in Table 2.

**Table 2:** EGFR mutations detected by SNapShot, qPCR and NGS

ID	Tumor cell content (%)	Identified mutations				
		qPCR	SNaPShot	NGS		
				DNA change	Designation	Total Coverage (%, number of reads)
BM 40	60	*T790M*	*T790M*	*c.2369C > T,*	*T790M,*	40 % (141),
		*L858R*	*L858R*	*c.2573 T > G*	*L858R*	23 % (60)
BM 49	25	*L858R*	*L858R*	*c.2573 T > G*	*L858R*	38 % (321)
BM 65	10	*L858R*	none	Not detected by automatic analysis		
BM 71	6	*DEL 19*	*DEL 19*	*c.2236_2250delGAATTAAGAGAAGCA*	*delELREA*	73 % (2467)
BM 72	40	*L858R*	*L858R*	*c.2573 T > G*	*L858R*	61 % (554)
BM 75	48	*G719X*	*G719X*	*c.2155G > A,*	*G719S,*	24 % (63),
				*c.2327G > A*	*R776H,*	58 % (28),
BM 76	2	*DEL 19*	none	Not detected by automatic analysis		
BM 82	24	*L858R*	*L858R*	*c.2573 T > G*	*L858R*	25 % (129)
BM 101	4	*DEL 19*	*DEL 19*	*c.2185G > A*	*G729R,*	6 % (10),
				*c.2235_2249delGGAATTAAGAGAAGC*	*delKELREA,*	26 % (48),
				*c.2260A > C*	*K754Q,*	27 % (50),
				*c.2279 T > C*	*L760P,*	6 % (11),
BM 145	14	*DEL 19*	*DEL 19*	Not detected by automatic analysis		
BM 150	56	*DEL 19*	*DEL 19*	Not detected by automatic analysis		
BM 157	72	*L858R*	*L858R*	*c.2573 T > G,*	*L858R*	74 % (792),
BM 159	3	*L858R*	none	*Not detected by automatic analysis*		
				*c.2125G > A,*	*E709K,*	7 % (36),
BM 160	5	*L858R*	none	Not detected by automatic analysis		

The NGS approach followed by an automatic analysis using the default 5 % variant frequency/5× variant coverage threshold allowed the identification of only 10 mutations from the SNaPshot/qPCR panel. The other five mutations were present in the NGS readings failing to reach the default calling thresholds - Table 3 but were picked out by manual reviewing known hotspots. Globally while the mutations identified using default variant calling parameters had an average mean read depth of 723 +/- 312× the missed five mutations had a mean coverage of 576 +/-398×.

**Table 3:** NGS run metrics for false negative results samples

Id	Mutation	Mean read depth	Hotspot coverage	Hotspot relative coverage	Variant frequency (%)	Variant coverage	Tumor cell content (%)	DNA concentration (ng/μl)
BM 65	*L858R*	1039	435	0.41	4.6	20	10	5.05
BM 76	*DEL19*	891	1028	1.15	2.9	29	2	22.9
BM 145	*DEL19*	36	2	0.05	100	2	14	4.91
BM 159	*L858R*	480	322	0.66	2.2	7	3	11.9
BM 160	*L858R*	436	299	0.68	4.4	13	5	0.22

However the NGS identified other sequence variations: three insertions *(c.2310_2311insCAC, c.2317_2319dupCAC, c.2315_2320dupCCCACG)*, five point mutations *c.2327G > A (R776H), c.2125G > A (E709K), c.2260A > C (K754Q), c.2185G > A (G729R), c.2279 T > C (L760P)*.

There were also 58 silent variants -54 *c.2361G > A (Q787Q)*, and one of each: *c.339C > T (Y113Y), c.876G > A (V292V), c.2430C > T (G810G), c.2319C > T (H773H)*.

An estimate of workload and costs are shown in Table 4 – initial DNA extraction not included (around 5 man hours workload and 24 h for final DNA solution at a cost of 10 euros/sample).

**Table 4:** Estimated workload and cost for EGFR mutation assessment using SNapShot, qPCR and NGS

	SNaPshot + Fragment analysis	q PCR	NGS
Price/10 samples	450 Euro	1300 euro	2500 euro
Price per gene/hotspot interogated	45	130	5
Workload/10samples	7 h	3 h	18 h
Time to result	48–72 h	5 h	72 h

## Discussion

Nowadays EGFR mutation assessment has become the standard approach in non-small cell lung cancer management and is available in most countries. There are many techniques available but there is no strong consensus on optimum approach and further complications may arise from the need for simultaneous multiple target testing (such as larger DNA quantities).

Presently EGFR mutation testing relies on amplification/sequencing approaches while other actionable abnormalities such as the *EML4-ALK* translocations are usually detected by fluorescence in situ hybridization or immunohistochemical analysis [10]. More targets are expected to become relevant as new drugs emerge *(PI3K)* [11, 12] or existing ones expand their initial indications (BRAF, HER2) [13, 14]. Considering these developments the need for not only precise and sensitive but also cost effective and scalable detection methods becomes acute as some of these mutations affect less than 1 % from the NSCLC patients.

False negative results may occur as a consequence to poor quality samples or faulty technique implementations but also due to the intrinsic limitations of each technique; post-analytical data interpretation should take into account these inherent limitations. This is especially relevant to NGS approaches as results depend on a long chain of serial technical steps and therefore multiple check-points should be implemented, each one with specific minimal quality parameters.

The aim of our study was to compare three widely available detection methods in order to identify potential causes for discordant results and to define minimal quality parameters that need to be attained for a high confidence result.

Our data suggest that all three methods return concordant results for high tumor cell content samples (over 15 %); for these samples qPCR and SNaPshot were implemented without any particular technical difficulty and for NGS a minimum mean coverage of 300× proved sufficient to detect relevant mutations.

Differences emerged when low tumor cell content samples were considered (25 samples - 39 % of total samples) with a mean tumor content of 5.9 % +/- 3.8 %. – SNaPshot approach failed to detect four mutations out of seven. NGS approach required careful data analysis and post hoc variant calling filter for five low tumor cell content samples. These results are consistent with published data supporting tumor cell content sensitivity thresholds – around 1 % for qPCR [15], 10 % for SNapShot [16] and 5–20 % for IonTorrent depending on sample type [9] This may be particularly important as real life medical practice increasingly relies on minimally invasive diagnostic approaches - as it is the case for EBUS-TBNA or other techniques that usually provide small core or cytology samples [5]. Macro-dissection may improve tumor cell content but still requires the presence of large enough tumor spots. Microdissection may yield better results but require specific equipment and may significantly add to workload and total cost as skilled manpower and specific equipment that are usually expensive. Although our data showed no difference between bronchial biopsies and surgical specimens in terms of tumor cell percentage it should be noted that banked and standard practice biopsy specimens may differ in terms of tissue quality.

Despite NGS high overall mean coverage, read quality and uniform read distribution there were some cases requiring careful data consideration. Extracted DNA quality may explain one false negative result - one sample with low mean coverage (36×) despite a 14 % tumor cell content. This was probably due to higher DNA fragmentation (only 62 bp mean read length [17]. Four false negative results were generated by using the default variant frequency threshold of 5 %. While this threshold is useful to filter out erroneous variant calls it should be cautiously applied to known hotspots at least for low coverage and/or low quality samples. For these positions high sensitivity should probably be prioritized over positive predictive power possibly by implementing an adaptive variant caller strategy taking into account tumor cells content and allowing for intra tumor molecular heterogeneity. In these cases a second approach (for ex qPCR) may be necessary in order to confirm results before validating the data for clinical use.

Effective NGS implementation requires good quality DNA preferably from a tumor rich sample, careful amplification and library construction, and careful post analytic data interpretation. Moreover according to our experience around 10 % of samples required additional runs and 2 % of the NGS libraries were recreated due to low coverage.

Despite these constraints the NGS approach returned richer data due to massive parallel analysis power and various additional sequence variations were identified, including five rare point mutations - *c.2327G > A (R776H), c.2125G > A (E709K), c.2260A > C (K754Q), c.2185G > A (G729R), c.2279 T > C (L760P)*.

There is little information available on *c.2327G > A (R776H)* mutation clinical significance and mainly from single case reports. Apparently *c.2327G > A* mutations may occur as germline variants [18] and may be associated to squamous differentiation [19]. Our case associated the *c.2327G > A* and *c.2155G > A (G719S)* mutations - an interesting occurrence given the low individual frequency for each. This association has been previously reported as retaining in vitro sensitivity to gefitinib and erlotinib [20].

The *c.2125G > A (E709K)* substitution is not a frequent occurrence and has been associated to tyrosine kinase inhibitors sensibility (TKI) [4]; response may be less favorable when compared to exon 19 and 21 mutations [21].

The *c.2185G > A (G729R)* has been reported in lung adenocarcinoma individual cases [22] and *G729E* in head and neck squamous cell carcinoma [23]; available results mentioned progressive disease.

Similarly the *c.2279 T > C (L760P)* substitution has been mentioned as an unique finding in a case report of a lung adenocarcinoma patient of Asian descent [24]; there was favorable clinical and imagistic response following icotinib therapy.

To our knowledge no relevant data was published concerning the *c.2260A > C (K754Q)* mutation. This mutation occurred in association with an exon 19 deletion and two other substitutions - *c.2185G > A (G729R)* and *c.2279 T > C (L760P)*. This particular sample also associated two other silent mutations *c.876G > A (V292V)* and *c.2361G > A (Q787Q)*.

The NGS detected exon 20 insertions involved two frequently affected sites - codons 771 and 774 [25]. Despite some clinical data suggesting that at least some exon 20 insertions might associate susceptibility to certain TKI agents [26] this mutation type is generally considered as non-sensitizing at least to gefitinib and erlotinib [27]. Still the *c.2322G > CCACGTG (V774_C775insHV)* which was detected in our sample pool was reported as associating partial response to chemotherapy and gefitinib [28].

In short there were five samples (~8 %) for which NGS specific data was clinically significant although not necessarily inducing management decision changes - such is the case for two of the exon 20 inserts.

NGS also identified a rather large number of single nucleotide polimorphisms (SNP). Codon 787 was most frequently involved - the *c.2361G > A (Q787Q)* polymorphism was found in 54 samples (84.3 %). This polymorphism have previously been reported for lung [29, 30], head and neck [31] and colorectal adenocarcinomas [32]. Available data attaches little physiological significance to *c.2361G > A* as it seems to be mainly a germline mutation and occurrence frequency is comparable for cancer and healthy subjects [32]. Still there is data suggesting potential association between lung adenocarcinoma microcystic histology pattern and the presence *c.2361G > A* polymorphisms [33]. From the therapeutic point of view there are data suggesting better response to gefitinib therapy for head and neck cancer cells harboring heterozygous *c.2361G > A* [31], possibly explained by abnormal splicing [31]. For our study sample pool there were 30 (55 %) cases exhibiting heterozygous *c.2361G > A*. Unfortunately TKI response data is not available for our study group and no further analysis is possible.

To our best knowledge the other four polymorphisms *c.339C > T (Y113Y), c.876G > A (V292V), c.2430C > T (G810G), c.2319C > T (H773H)* have not been reported as exerting any significant physiological effect.

These results support the idea of targeted approaches such as qPCR being the first choice for low tumor content samples at least if only one mutation hotspot is targeted. This method is accurate, sensitive, simple and applicable to FFPE samples [5]. Comparing the two targeted methods alone SNaPshot showed similar results to qPCR at a significantly smaller price per sample and somewhat higher workload [34] but for low tumor cell content samples it may lack sensitivity. The decision of using one method or another should probably be made considering additional factors - such as existing equipment, available expertise and laboratory profile. And if *in house* methods are used, a careful validation procedure should be done [35].

NGS returned comparable results to targeted methods in terms of overall accuracy. False negative NGS results may be diminished by using various tumor cell enrichment procedures and careful and context sensitive data interpretation. In some cases alternative testing methods and/or sample reruns may be necessary.

NGS low tumor cell content issues seems to be counterbalanced by ensuring an adequate depth of reading coverage both in terms of mean read depth and also local hotspot coverage and careful data analysis. The capacity of detecting rare and previously unknown mutations sometimes of clinical significance makes it a valuable scientific tool. Although rare mutations may eventually be included in targeted panels significant cost issues may be expected.

From a strict financial perspective NGS is the most expensive technique. If the scope is widened and multiple actionables are simultaneously considered (for example EGFR*,* BRAF*, KRAS*) cost becomes equivalent to PCR based methods. In order to reach the SNaPshot cost level at least 5 to 7 different hotspots must be assessed.

Flexibility and capacity of assessing multiple targets in one run make NGS a valuable tool taking into account the fact that not only EGFR but other genes are emerging as potential targets in lung cancer management.

## Conclusion

All three methods return similar results for high tumor content samples. Careful post analytic interpretation of results is necessary especially for low tumor content samples. Tumor cell sample enrichment techniques may be useful. NGS is able to generate a comprehensive mutational profile albeit at a higher cost and workload. Result interpretation should take into account not only general run parameters such as mean read depth but also relative coverage and read distribution; currently there is an acute need to define firm recommendations/standards concerning NGS data interpretation.

## Abbreviations

DNA: Deoxyribonucleic acid
EBUS: Endobronchial ultrasound
EGFR: Epidermal growth factor receptor
Ex: Exon
FFPE: Formalin, fixed paraffin, embedded
FWD: Forward primer
Min: Minutes
NGS: Next generation sequencing technique
NSCLC: Non squamous non, small cell lung cancer
PCR: Polymerase chain reaction
PGM: Ion personal genome machine system
qPCR: Quantitative polymerase chain reaction
REV: Reverse primer
Sec: Seconds
SNP: Single nucleotide polimorphisms
TBNA: TransBronchial Needle Aspiration
TKIs: Tyrosine Kinase Inhibitors
